# Working towards universal health coverage: a qualitative study to identify strategies for improving student enrolment for the pre-service training of nurses, midwives and community health workers in Nigerian health training institutions

**DOI:** 10.1186/s12960-021-00560-9

**Published:** 2021-02-12

**Authors:** Ekechi Okereke, Babatunde Ahonsi

**Affiliations:** 1 Population Council, 16 Mafemi Crescent, Off Solomon Lar Way, Utako, Abuja, Nigeria; 2 UNFPA China, 1-161 Tayuan Diplomatic Office Building, Beijing, China

**Keywords:** Student enrolment, Pre-service training, Frontline health workers, Universal health coverage, Nigeria

## Abstract

**Background:**

Student enrolment processes and practices can affect the quality of pre-service training programmes. These processes and practices may have serious implications for the quality and quantity of students within health training institutions, the quality of education for prospective health workers and consequently health workforce performance. This study assessed current student enrolment processes and practices for nurses, midwives and community health workers within health training institutions in two Nigerian states, so as to identify strategies for improving student enrolment for these key cadres of frontline health workers.

**Methods:**

This study was carried out in Bauchi and Cross-River States, which are the two Human Resources for Health (HRH) project focal states in Nigeria. Utilizing a qualitative research design, 55 in-depth interviews and 13 focus group discussions were conducted with key stakeholders including students and tutors from pre-service health training institutions as well as policy-makers and public sector decision-makers from Ministries of Health, Government Agencies and Regulatory Bodies. Study participants were purposively sampled and the qualitative data were audio-recorded, transcribed and then thematically analysed.

**Results:**

Study participants broadly described the application process to include the purchase, completion and submission of application forms by prospective students prior to participation in entrance examinations and oral interviews. The use of ‘*weeding examinations’* during the student enrolment process, especially in Bauchi state, was identified as a useful quality assurance mechanism for the pre-service training programmes of frontline health workers. Other strategies identified by stakeholders to address challenges with student enrolment include sustained advocacy to counter-cultural norms and gender stereotypes vis-à-vis certain professions, provision of scholarships for trainee frontline health workers and ultimately the development as well as effective implementation of national and state-specific policy and implementation guidelines for the student enrolment of key frontline health workers.

**Conclusion:**

While there are challenges which currently affect student enrolment for nurses, midwives and community health workers in Nigeria, this study has proposed key strategies which if carefully considered and implemented can substantially improve the status quo. These will probably have far-reaching implications for improving health workforce performance, population health outcomes and efforts to achieve universal health coverage.

## Background

Sub-Saharan Africa has an acute shortage of healthcare workers [1, 2], including key frontline health workers such as nurses, midwives and community health workers. However, as part of health workforce development, scaling up health worker education and training is important. There were commitments during the Second and Third Global Forum on Human Resources for Health [3, 4] which were reinforced more recently at the Fourth Global Forum on Human Resources for Health in 2017, to scale-up health worker education/training. Such commitments and efforts to scale-up health worker education and training as part of health systems strengthening are necessary in order to achieve adequate numbers and the right quality of healthcare workers, especially for countries within sub-Saharan Africa.

In many states in Nigeria, there are health training institutions which are owned and managed by the Federal Government, usually through Federal Universities such as the University of Calabar in Cross-River state and Abubakar Tafawa Balewa University in Bauchi state. Universities offer 5 years Bachelor of Nursing undergraduate programmes but Postgraduate programmes for nursing professionals (Masters’ and PhD) also exist [5]. Some states have established stand-alone state-owned Colleges and Schools of Nursing and Midwifery, which offer basic nursing programmes, designed to train students to become registered nurses after three years [6]. Nursing graduates who wish to practise in Nigeria are required to take licensure examinations before becoming registered nurses with the Nursing and Midwifery Council of Nigeria [5].

Nurses and midwives have a broad range of responsibilities and contributions to health systems and as key frontline health workers, have critical roles to play in efforts to achieve universal health coverage. In particular, nurses ensure high quality healthcare, engage in health promotion and promote patient safety. They are involved in the management of noncommunicable diseases as well as the prevention, treatment and control of communicable diseases in both primary and non-primary healthcare settings. Nurses, midwives and obstetricians provide antenatal, intrapartum and postnatal services for women of reproductive health age, in addition to neonatal and child health care services for children and adolescents. Nurses also provide healthcare services during clinical emergencies, e.g. accidents, and contribute to responses for epidemics, disasters and humanitarian crises [7].

Some states in Nigeria have also established Colleges of Health Technology to train community health workers as part of efforts to increase their numbers of frontline healthcare workers. According to National Guidelines for primary healthcare systems in Nigeria, junior community health extension workers (JCHEWs) should promote the community’s participation in health-related activities, conduct home visits, undertake clients’ follow-up as well as identify and then register pregnant women for antenatal care (ANC). The JCHEWs should spend about 90% of their time working within communities but in addition they should provide some health facility-related services [8, 9]. JCHEWs are trained for about 2½ years on the same skills as community health extension workers (CHEWs), but the secondary school certificate scores required for admission for JCHEW training are lower than the scores required for entry into CHEW training [6]. Furthermore, a JCHEW requires two additional years of training in Colleges of Health Technology to be certified as a CHEW. CHEWs train for 3 years to provide basic health services in primary healthcare facilities and within communities [6]. CHEWs should spend less time (~ 40% of their time) working within communities and more time working in health facilities, managing patients and attending to clients according to clinical protocols [9]. Both CHEWs and JCHEWs do not receive competency-based midwifery training during their pre-service training in Colleges of Health Technology [6, 10]. Lastly, community health officers (CHOs) have more managerial responsibilities in health facilities, but also carry out similar community-based and facility-based tasks as the CHEWs [9].

The World Health Organization (WHO) in its *Global Health Observatory Data Repository* reports that in Nigeria there were 116,454 community health workers, 110,105 nursing personnel and 120,870 midwifery personnel as at 2018 [11], which is inadequate for the country’s population estimated close to 190 million [according to WHO’s Global Health Observatory]. Nigeria is listed as one of the countries across the world which have a critical shortage of health workers [12, 13] and specifically in 2018 it was listed among countries with the largest shortages of nursing professionals (in numerical terms), alongside Bangladesh, India and Pakistan [7]. Across Nigeria, there are many challenges which have given rise to the shortage (and maldistribution) of frontline health workers, especially from where they are most required [14]. The effective delivery of maternal, newborn and child health services especially at primary healthcare level is largely dependent on the availability and equitable distribution of well-trained and skilled health workers [15] including nurses, midwives and community health workers. According to the 2018 Nigeria Demographic and Health Survey (NDHS), approximately two-thirds of pregnant women in Nigeria utilize antenatal care services but only about 40% deliver with assistance from skilled birth attendants. Furthermore, Nigeria has a maternal mortality ratio of 512/100,000 live births, infant mortality ratio of 67/1000 live births and under-5 mortality ratio of 132/1000 live births [16]. There are however some key government initiatives in Nigeria, such as the Primary Healthcare Under One Roof (PHCUOR), which have placed more emphasis on primary healthcare service delivery and the frontline health workforce [17].

**Fig. 1:**
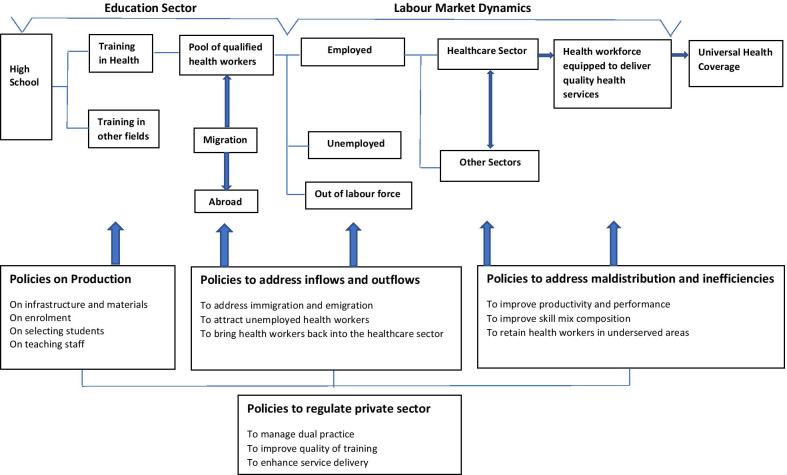
Health labour market framework and policy levers for attaining universal health coverage (UHC). Adapted from Sousa A, Scheffler RM, Nyoni J, Boerma T. A comprehensive health labour market framework for universal health coverage. *Bulletin of the World Health Organization*. 2013; 91:892–4

Nigeria’s National Strategic Health Development Plan (2018 – 2022) emphasizes the Government’s determination to strengthen health workforce development [18], including strengthening health training institutions. The development of the National Strategic Health Development Plan is in tandem with efforts to achieve universal health coverage by governments around the world. Regulatory bodies in Nigeria are mandated to provide guidance to different health training institutions on student enrolment for key frontline health workers in Nigeria, i.e. nurses, midwives and community health workers. Anecdotes suggest that the steps in the student enrolment process include: the sale of application forms, selection of students, registration and then orientation for students. A systematic analysis of student enrolment processes and practices for Colleges/Schools of Nursing and Midwifery (for nurses and midwives) as well as Colleges of Health Technology (for community health workers) in Nigeria is however currently lacking. Analyses on student enrolment processes and practices can contribute towards the development of effective policies for the production of healthcare workers and ultimately help to achieve universal health coverage as illustrated by the Health Labour Market (HLM) framework [19] (Fig. 1).

The human resources for health (HRH) project, implemented by Population Council and the World Health Organization in Nigeria, carried out an assessment of the student enrolment processes and practices for nurses, midwives and community health workers in public health training institutions. The study assessed current student enrolment processes and practices for nurses, midwives and community health workers within health training institutions in two Nigerian states, so as to identify strategies for improving student enrolment for the pre-service training of these key cadres of frontline health workers. The HRH project is a health system strengthening project which has an ultimate goal to further develop the Nigerian frontline health workforce. Within the HRH project, frontline health workers refer to nurses, midwives and community health workers, i.e. community health extension workers, junior community health extension workers and community health officers [9]. The HRH project collaborated with the Nursing and Midwifery Council of Nigerian (NMCN), which is the regulatory institution for nurses and midwives in the country as well as the Community Health Practitioners’ Registration Board of Nigeria (CHPRBN) which is the regulatory institution for community health workers in Nigeria [9, 20, 21]. The NMCN and CHPRBN, as regulatory institutions typically liaise with health training institutions during the development and upgrade of training curricula, during the student enrolment process as well as to ensure the enforcement of standards for graduation, indexing and licensure of these key cadres of healthcare workers, i.e. nurses, midwives and community health workers in Nigeria.

## Methods

### Study sites

This study was carried out in Bauchi state in the north-eastern part of Nigeria as well as in Cross-River state within the Southern part of the country*.* Both states were selected for this study because these are HRH project focal states, but in addition, collecting and then contrasting data from both states which are geographically as well as culturally different should provide valuable insights about the student enrolment processes and practices within publicly owned health training institutions for nurses, midwives and community health workers.

### Study design, study population and sampling strategy

This assessment adopted a qualitative research design utilizing in-depth interviews and focus group discussions for data collection. The in-depth interview guides for stakeholders and managers of the healthcare system focused on eliciting answers for questions on policies, guidelines and procedures for enrolling students while the in-depth interview guides for students were designed to elicit answers for questions focused on the experiences of students during their enrolment into health training institutions. Focus group discussion guides for healthcare managers were also designed differently from the focus group discussion guides for students and tutors. There was a total of 55 in-depth interviews with students and key stakeholders, i.e. 30 in Bauchi and 25 in Cross-River states. Persons interviewed for the assessment include students who were enrolled in public pre-service health training institutions in Bauchi and Cross-River states, tutors who were employed in public pre-service health training institutions in Cross-River and Bauchi States as well as policy-makers, public sector decision-makers, senior healthcare managers from Ministries, Government Agencies, Regulatory Bodies and relevant Professional Associations. In addition, there were a total of 13 focus group discussions with healthcare managers, tutors and students (8 in Cross-River and 5 in Bauchi). All eleven publicly owned health training institutions in Bauchi and Cross-River States were involved in the assessment. This consisted of seven health training institutions in Cross-River State (School of Nursing Itigidi, School of Nursing Ogoja, School of Nursing Calabar, School of Post-Basic Midwifery Ogoja, School of Midwifery Obudu, School of Post-Basic Midwifery Calabar and the College of Health Technology Calabar) and four health training institutions in Bauchi State (School of Nursing Bauchi, School of Basic Midwifery Bauchi, School of Community Midwifery Bauchi and the College of Health Technology Ningi). The public health training institutions involved in the study are located mainly in urban and semi-urban areas within Bauchi and Cross-River states. Public health training institutions typically offer the majority of vacancies to prospective applicants to become nurses, midwives and community health workers when compared to private health training institutions as there are more public health training institutions than private health training institutions for nurses, midwives and community health workers. All the public health training institutions included in the study have accreditation from the relevant regulatory bodies, i.e. Nursing and Midwifery Council of Nigeria and the Community Health Practitioners’ Registration Board of Nigeria [20, 21]. A purposive sampling approach was employed to select key informants for the in-depth interviews as well as for the focus group discussions. The purposive sampling technique was appropriately selected to ensure that all study participants were well-suited to discuss and offer valuable insights to address the objectives of the study.

### Data collection and data management

Interviewers who had prior experience conducting in-depth interviews or facilitating focus group discussions were recruited for the data collection process. For quality assurance purposes, the interviewers were appropriately trained on the use of the in-depth interview and focus group discussion (FGD) guides. Interviewers for the study were also retrained on research ethics*.* Written informed consent was sought and obtained from each study participant prior to commencing the in-depth interviews and focus group discussions. Data collection for the assessment via in-depth interviews and focus group discussions took place in both Bauchi and Cross-River states in November and December 2018. The interviews and focus group discussions were recorded using tape recorders. Consent to be tape-recorded was sought and obtained from the study participants as part of the informed consent process. After the interviews, the interviewers transcribed the audio recordings verbatim and the transcripts and audio recordings were sent to Population Council Nigeria Country office for archiving. The transcripts were subsequently compared with the audio recordings to ensure completeness. A pre-determined coding framework was applied, and subsequently thematic analyses of the data were undertaken. The study team members provided assistance to categorize the qualitative data and regular meetings were held by the researchers to reach consensus on the interpretation of the data.

### Ethical considerations

Ethical approval was granted by Bauchi and Cross-River States’ Research Ethical Committees and Population Council’s Institutional Review Board (IRB) in New York, USA. The study was conducted based on set ethical guidelines and respondents were assured of the confidentiality of their responses. Written informed consent was secured from each study participants and copies of the signed consent forms were compiled and stored in Population Council’s Country office in Abuja.

## Results

**Fig. 2:**
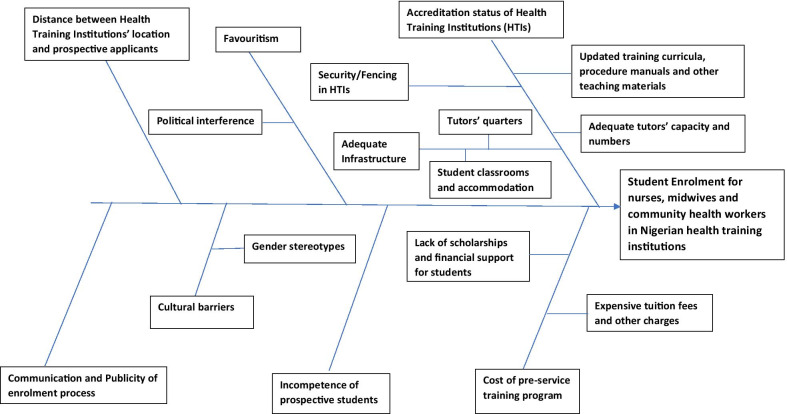
Fishbone diagram outlining factors which influence/affect the student enrolment process in Nigerian health training institutions

The results for the study are presented in two sections—Section A outlines the different categories for the student enrolment process in Nigerian health training institutions; starting with the ‘Application process’ and ending with ‘Publicity for the student enrolment process’. Section B outlines the different categories and sub-categories with respect to challenges which can affect/influence the student enrolment process in Nigerian health training institutions. For Section B, the key categories identified from the study include accreditation status of health training institutions, cost of the pre-service training programme, political interference and favouritism, cultural barriers, distance between health training institutions’ location and prospective applicants, communication and publicity of enrolment process as well as incompetence of prospective students. Some of these categories have sub-categories. A fishbone diagram (Fig. 2) outlines the interconnectivities of these challenges to the student enrolment process for nurses, midwives and community health workers in Nigerian health training institutions.

### Section A: Student enrolment processes and practices in health training institutions

#### The Application process

The application process is slightly different for the various programmes, for example within the College of Health Technology, the process for applying to become a community health extension worker (to obtain a diploma) is different for a prospective student applying to be a junior community health extension worker (to obtain a certificate). Also, applicants to the School of Nursing go through a different process in contrast to prospective students applying to the School of Post-Basic Midwifery. However, stakeholders mentioned that the application process is done to help select the best possible candidates for the different programmes in the various health training institutions.

“The general purpose is to ensure that we have students that are capable of going through the program and of right gender mix so that we can meet the needs of the health sector in the state.”

**Stakeholder, IDI, Cross-River**

Broadly speaking, the application process starts with the sale of forms either at the Ministry of Health or by the health training institutions within each state. Some stakeholders indicated that announcements are typically made to publicize the start of the sale of forms. Subsequently, a date for the written entrance examination is fixed. Applicants who obtain the minimum pass mark of 50 or above are subsequently shortlisted and invited for an oral interview. Applicants who are invited for the oral interview are requested to come with their credentials, so that the interview panel can confirm that the applicants possess the minimum 5 Credits in Physics, Chemistry, Biology, English language and Mathematics. After the oral interviews, the raw scores are submitted to the management of the School/College. The scores from the oral interview and the scores from the entrance examination are combined and the School/College selects the best applicants based on these results.

“Buy the application form, then complete it and submit for them to give you an examination number. That number is what you use on the examination date to write the exams. The management of the school will then sit down and put the marks obtained in the entrance examination alongside the marks for the oral interview and then they select based on the performance of the students. Such persons that have been selected will be admitted into either School of Nursing or School of Basic Midwifery or College of Nursing and Midwifery. That's the process…”

**Stakeholder, IDI, Bauchi**

However, for students vying to enter the School of Post-Basic Midwifery, there is the requirement that such applicants must be registered nurses with the Nursing and Midwifery Council of Nigeria with current practising licenses. Such applicants buy the application forms following the advertisement for the sale of forms and then are invited for a selection interview. During interviews, applicants’ credentials are inspected to confirm that they are registered nurses. Applicants are also asked oral questions as part of the interview process. Successful applicants are subsequently issued admission letters by the Ministry of Health.

“…because our school is a post basic institution, there must be evidence that they [applicants] have been registered following their previous training at a School of Nursing and have their certificates…there is a selection interview and when that is done, successful applicants are sent to the different institutions, we have two schools of Post-Basic Midwifery in Calabar and Ogoja”

**Stakeholder, IDI, Cross-River**

The application process for the College of Health Technology follows a similar pattern. Stakeholders mentioned that advertisements are done and then adequate time is given for interested candidates to buy the forms. An aptitude test and oral interviews are used to select successful applicants by combining scores from both the tests and interviews.

“After the exams I had an interview…they made sure we passed through different processes [so that] they will be sure of the people they are admitting…”

**Student, FGD, Cross-River**

#### The selection process and the involvement of regulatory bodies

Regulatory bodies are involved in the selection process within health training institutions and this includes determining the criteria for selection of students to be enrolled. Study participants reported that state-owned health training institutions give preference to applicants who are state indigenes, but with some provisions for non-indigenes. In addition, applicants from different local government areas within the state are given due consideration during the selection process to ensure that all the local government areas have representation in these state-owned health training institutions. However, both Federal and state-owned health training institutions require that applicants have at least 5 Credits in English, Mathematics, Biology, Physics and Chemistry as a minimum requirement.

“…nationwide criteria for health institutions is that somebody must have 5 Credits in English, Mathematics, Biology Physics and Chemistry, this is the criteria in terms of O'level qualification…”

**Stakeholder, IDI, Bauchi**

“So, for the selection we use merit…and also the quota system...we even give percentages to other catchment areas, that is our neighbouring states at least about 5 percent”

**Stakeholder, IDI, Bauchi**

“We admit mainly from within our state because we want our indigenes who enroll to become healthcare workers; we also ensure that the different local government areas in the state are given adequate consideration…there are however students from outside our state studying here!”

**Stakeholder, IDI, Cross-River**

The number of students to be enrolled depends on the regulations and directives from the relevant regulatory bodies, and the number is also determined by factors such as the accreditation status, infrastructure and resources (including the human resources, e.g. number of tutors) available in the health training institutions. For the Schools/Colleges of Nursing and Midwifery, the Nursing and Midwifery Council of Nigeria (NMCN) requires that 50 students be indexed each year, however this number can sometimes be more or less than 50 students, depending on discussions and directives from the NMCN. Policy-makers and heads of health training institutions (especially in Bauchi state) mentioned that although Colleges/Schools of Nursing and Midwifery typically have a benchmark of 50 students per year which should be indexed with the NMCN, there is the understanding that some students may fail examinations and maybe drop out from the institution for various reasons. Hence the initial number of students admitted could be up to 100 students and after ‘weeding examinations’ the required number of ‘qualified’ students are then indexed with the regulatory bodies as required by standard regulation. ‘Weeding examinations’ are tests which are organized by some public health training institutions, especially the College of Nursing and Midwifery in Bauchi state, to further assess the capacity of the students who were initially enrolled so that the very best among the students can be selected for subsequent indexing with the relevant regulatory bodies.

**“**It depends on what the Council approves, if the school has full accreditation and has the facilities on ground, Council can ask you to enroll up to one hundred per entry or maybe forty or fifty depending on the facilities and the resources. But if you have partial accreditation, they can ask you to take maybe thirty, forty or fifty; so, it depends on what the Nursing and Midwifery Council approves or your accreditation status with the Council.”

**Tutor, FGD, Cross-River**

“…what Nursing and Midwifery Council does is that they have regulations; they have standards which they expect should be followed…”

**Stakeholder, IDI, Bauchi**

Many study participants complained about interferences, including interferences from politicians, during the selection process and some stakeholders indicated that the ‘weeding examination’ is done to remove weak students and those who may not be able to cope during the programme. Irrespective of the number of application forms sold to prospective applicants when the student enrolment process begins, the ‘weeding examination’ is usually done so that health training institutions only index competent students with the NMCN. ‘Weeding examinations’, as the final step in the selection process, are typically conducted by health training institutions about 3 to 6 months after the initial admission of the students to enable the final selection of students to be completed before the end of the first year.

“The final measure for enrollment is our internal examination which is not interfered with by anybody and is known as ‘weeding examination’…we hope that anybody that is able to make it through the ‘weeding examination’ will be able to cope during the program”

**Stakeholder, IDI, Bauchi**

“So, after ‘weeding examination’ then we will select the best among them to get the required number that we need… “

**Stakeholder, IDI, Bauchi**

For Colleges of Health Technology, the number of students to be indexed with the regulatory body, i.e. Community Health Practitioners Registration Board of Nigeria (CHPRBN) is dependent on discussions between the regulatory body and the respective institutions in each state. Some stakeholders mentioned that because of the high demand for CHEWs, the regulatory body could allow more students to be indexed per year per College of Health Technology. It was reported that some JCHEWs after completing their certificate programme, advance to the Diploma programme to become CHEWs and the Colleges of Health Technology make provisions for such applicants. For example, if a College of Health Technology is given permission to index 75 students as CHEWs by the regulatory body, the institution may give 25 slots to applicants who have completed their certificate programmes as JCHEWs and give fifty slots to applicants who have just completed their senior secondary school education and have the minimum qualifications.

### Scholarships and ‘bonding’ agreements

Many students complained about not getting financial support in the form of scholarships from the government for nursing, midwifery and community health worker programmes, even though scholarships might be available for other health professionals, e.g. doctors/physicians. It was mentioned by study respondents in Bauchi state that allowances are given to students who are successful in the ‘weeding examinations’ and are paid to support such students through the programme till graduation. Policy-makers reiterated that these allowances are given/paid to students who have been indexed with the regulatory bodies. Such indexed students are given ‘bonding agreements’ to sign and are thus government-funded trainee nurses or midwives. These trainee frontline health workers, after being indexed with the regulatory body, are employed into the Civil Service and placed on the entry level position for health workers as a government employee within the public service in Nigeria.

“…when you fill that bond, it is like they will pay you and after you finish as a student you will serve the state government for like two years, so even if you are thinking that okay after this school, I am going to go to another school but because of that bond, you will have to serve for two years…they only give their indigenes because they want them to serve their state…”

**Student, FGD, Bauchi**

### Student orientation and induction

Student orientation and induction is necessary for students in health training institutions when they start their programme in the College/School of Nursing and Midwifery or at the College of Health Technology. For some institutions, it starts with registration as a student, involves introduction of the students to their tutors and other members of staff as well as familiarization of the students with the premises of the health training institution and its resources. In some institutions, there is a formal ceremony where staff and invited persons give short speeches to the students, explaining the courses on offer within the institution, facilities available within the institution’s premises including laboratories, libraries and computer rooms, the rules and regulations as well as future career prospects of students upon graduation. Many students expressed the usefulness of the orientation and induction while also describing their personal experiences during their student orientation and induction.

“…the day we resumed, that's the day we did our registration and started lectures; it was like after a week that they did orientation for us, they took us around the school, some of the lecturers introduced themselves, they told us the courses we were going to take, the rules and regulations guiding the school, they even took us to the library, telling us we can come and borrow some books and read; also we went to the computer station; they showed us all the offices in the school and who are staying there”

**Student, IDI, Bauchi**

### Publicity for the student enrolment process

Publicity for different stages of the student enrolment process is done through a number of channels. These include the mass media, i.e. television and radio, in addition to traditional means of using the noticeboards of Ministries, Departments and Agencies to raise awareness about the enrolment process. Churches, mosques and community announcements are also utilized as a means of disseminating information about the student enrolment process. More modern forms of communication such as using social media via Facebook, Twitter and WhatsApp were also mentioned as means of publicity for the student enrolment process. Some students indicated that word of mouth, phone calls and text messages from family and friends are common means of publicity about the student enrolment process.

“After writing the exams, if you pass they will still send text message for you to come for an interview...If you pass the interview, they will also send text message to you…I think this is much better than only placing the results on the noticeboards because if you don't have anybody to inform you that the results are out and on notice boards, they will do interviews and complete admissions and you don't even know…”

**Student, IDI, Bauchi**

“…we use the social media and equally send information to all other institutions, but at the state level a general advertisement is done, which is sent to all the local government headquarters and to health institutions; information is also sent to the churches…”

**Stakeholder, IDI, Cross-River**

### Section B: Challenges to the student enrolment process in health training institutions

#### Accreditation status of health training institutions

Health training institutions require accreditation from the relevant regulatory bodies to admit students to be trained as healthcare workers. There are a number of factors which determine a health training institution’s accreditation status. These include ‘Adequate infrastructure’, ‘Security/Fencing in Health Training Institutions’, ‘Adequate tutors’ capacity and numbers’ as well as ‘Updated training curricula, procedure manuals and other teaching materials’.

#### Adequate infrastructure

Stakeholders mentioned that the availability of some basic infrastructure is a minimum requirement for regulatory bodies to give accreditation to health training institutions. However, feedback received was that much more infrastructure is required within many health training institutions, including more hostels for students, staff quarters for staff as well as other basic amenities such as potable water and electricity which students who are enrolled require to comfortably embark on and complete their training programmes.

“…most of the schools don’t have facilities for staff to stay - no staff quarters…”

**Stakeholder, IDI, Cross-River**

“…to get accreditation as a health training institution, you need to have the physical structures on the ground. Apart from physical structures, you need to have the [required] equipment…”

**Stakeholder, IDI, Bauchi**

#### Security/fencing in health training institutions

A small proportion of students from both study states complained about the lack of security in their institutions. They argue that much more needs to be done to address insecurity, as students will not seek admission and families will not encourage their members to enrol into institutions, if they perceive that there is lack of security within a health training institution. In addition, stakeholders reported that full accreditation for health training institutions will most likely be secured after there is adequate fencing and good security in these institutions.

“We don’t have security in this school because there was a time when they posted us to teaching hospital, a thief entered our hostel…”

**Student, FGD, Bauchi**

“We had to complete the fencing for our College before the Nursing and Midwifery Council of Nigeria granted us full accreditation…”

**Stakeholder, IDI, Bauchi**

#### Adequate tutors’ capacity and numbers

Insufficient numbers of tutors can be a limiting factor for health training institutions to obtain and secure accreditation from the relevant regulatory bodies. Inadequate systems and structures for human resource development for tutors within health training institutions can affect the quality *and numbers* of tutors available to teach students enrolled into health training institutions. There were complaints from study participants that more tutors need to be employed and better support mechanisms for the development of tutors are required within health training institutions.

“…we need more tutors who are registered as nursing educators. You know that not having enough qualified tutors can affect our students’ training and the accreditation status of our school”

**Tutor, FGD, Cross-River**

#### Updated training curricula, procedure manuals and other teaching materials

Most tutors and policy-makers from both states, who were involved in the study emphasized that the lack of updated training curricula, procedure manuals as well as other teaching materials can affect the decision of regulatory bodies to grant accreditation to health training institutions. They argued that the lack of these training materials can affect student enrolment into health training institutions.

“Regulatory bodies especially the Nursing and Midwifery Council of Nigeria, will always confirm that each institution has updated training curricula before full accreditation is issued to any health training institution…”

**Stakeholder, IDI, Bauchi**

“Institutions need updated training curricula before seeking accreditation from the Council…these teaching materials are necessary to properly train our students when we admit them.”

**Tutor, FGD, Cross-River**

#### Cost of pre-service training programme

The entirety of costs which prospective students (and their family) may incur to enrol and train as healthcare workers does affect student enrolment into health training institutions. Study respondents identified two key sub-categories with respect to the cost of pre-service training programmes; these include ‘Expensive tuition fees and other charges’ and ‘Lack of financial support for students’.

#### Expensive tuition fees and other charges

Most students from both study states involved in the study complained about costly tuition and registration fees in their institutions; pointing out that this has contributed to some students dropping out of health training institutions. Students reported that they are sometimes asked to pay for ‘hidden fees’ which they do not understand and for which there may be no justification. However, some stakeholders who were interviewed reported that any additional fees levied on students is to enable the management of the health training institutions to internally generate funds to more adequately manage the Schools/Colleges. Study participants indicated that the high tuition and registration fees discourage prospective applicants and persons desirous of becoming frontline health workers from enrolling into health training institutions.

“…I understand what the school fees are meant for, but the other charges what does that entail?”

**Student, FGD, Cross-River**

#### Lack of financial support for students

Almost all students involved in the study from both states complained that many potential students are discouraged to apply to health training institutions because of the lack of financial support available to students. Scholarships by the state government are sometimes offered to students who are studying to be doctors in universities, but financial support are unavailable for students studying to be nurses, midwives and community health workers. Students mentioned that although some allowances are paid to students who have been indexed with the regulatory bodies, these allowances are sometimes not regular and may not be enough for students who come from poor households.

“When they cannot pay, they can't cope…some people have dropped out of nursing because of money…”

**Student, FGD, Cross-River**

#### Political interference and favouritism

A small number of students from both states openly complained during the study that favouritism exists within their institutions. They argued that families of applicants who have access to elected officials and other influential members of society or those who have relationships/friendships with staff members of health training institutions have better chances of securing admission. Students especially mentioned that favouritism sometimes affects the outcomes of oral interviews during the enrolment process within health training institutions. The authorities of the institutions involved in this study denied these allegations. But there were these small number of students involved in the study who insisted that political interference is a major problem which health training institutions encounter. Politicians push for their wards or relatives to be admitted, when in some instances such applicants may be less qualified or not even qualified for admission. Some stakeholders particularly from Bauchi state mentioned that due to the influence of political interference and favouritism, that is why the ‘weeding examinations’ are very helpful to eventually select the very best students to be indexed with the regulatory bodies. Study respondents complained that politicians and influential individuals who interfere with student enrolment into health training institutions usually do not take into consideration that frontline health workers are employed to preserve and save human lives, thus only the most competent applicants should be enrolled into health training institutions.

“Political pressure and interference should be limited for the sake of the people in the society since we are dealing with life. Let people understand that all we are striving to do is to make sure that things are done in the right way so that people's lives are saved…”

**Stakeholder, IDI, Bauchi**

“…somebody will tell you, ‘ah it was through this person I got admission, we had to go here before I got admission, my father did this, my father did that’. I've never heard anybody saying he got it without somebody helping.”

**Student, IDI, Bauchi**

### Cultural barriers

There are cultural barriers and norms which stakeholders indicated affect the enrolment of students, especially males, into health training institutions. Most men within northern Nigeria in particular will typically object to their wives, daughters and mothers to receive healthcare services from a male health worker. They will request for and insist that a female health worker should attend to female members of their family. These cultural barriers have affected the enrolment of especially male applicants into various health training institutions such as Colleges/Schools of Nursing and Midwifery, particularly in northern Nigeria.

“…for midwifery you know it's a feminine program here in the North, there's no where they are admitting men for midwifery…”

**Stakeholder, IDI, Bauchi**

“…because of religious and cultural norms in this part of the country, people are not comfortable with a male attending to a female patient in a facility. They will always want to have a female health worker attending to a female...”

**Stakeholder, IDI, Bauchi**

#### Distance between health training institutions’ location and prospective applicants

Many students, especially from the rural areas of both study states, complained about the stress which many persons experience during the student enrolment process due to the distance between health training institutions and applicants as a deterrent to many interested and qualified applicants. Some applicants travel from their villages and sometimes neighbouring states to complete different stages and requirements of the student enrolment process without proper accommodation or having someone to stay with.

“Actually, there is a lot of stress in purchasing the form and writing the examinations because some [applicants] are living far away…”

**Student, IDI, Bauchi**

“…it was very, very stressful. Because like myself, I normally will come down from the village and my village to this place, it's about a hundred and something kilometers.”

**Student, FGD, Cross-River**

#### Poor communication and inadequate publicity

There were complaints, especially from students, that there is inadequate information sharing, short advertisement periods and not enough publicity about the different steps of the student enrolment process to members of the public from Ministries of Health and health training institutions.

“You know so many people that really wanted to come into the profession didn't get the opportunity because the advert period is just too short…”

**Student, FGD, Cross-River**

#### Incompetence of prospective students

The incompetence of some students is a challenge which affects student enrolment. Study participants indicated that although some applicants may have the minimum requirements of at least 5 credits in core science subjects, they may be unable to demonstrate the relevant capacity required by students who wish to enroll into health training institutions. Thus, even if aspiring students pass through both the written entrance examination and oral interviews, ‘weeding examinations’ are still used in some health training institutions, especially in Bauchi state, as an extra layer of quality control to ensure that only students who are qualified and likely to successfully complete the training programme are selected and subsequently indexed with the regulatory bodies.

“…you can pass the exam but on getting to the interview, you may fail the interview, automatically you will not be admitted…”

**Student, FGD, Cross-River**

“…the ‘weeding examination’ ensures that weak students are removed and only competent students continue on the program”

**Tutor, FGD, Bauchi**

## Discussion

This research study set out to evaluate current student enrolment processes and practices in two states (Bauchi and Cross-River) within Nigeria as well as identify key challenges, with the goal to provide recommendations to improve student enrolment, especially for nurses, midwives and community health workers in publicly funded health training institutions. Student enrolment processes and practices in health training institutions influence the quality and quantity of students [22], thus if these processes are strengthened, the health workforce as well as the health system would consequently be strengthened. Effective strategies and guidelines for student enrolment into health training institutions for frontline health workers should be part of HRH planning and contribute to policies for the production of healthcare workers. The health labour market framework (Fig. 1) depicts student enrolment and admission into health training institutions as crucial for generating a pool of qualified health workers and a health workforce which is equipped to deliver quality health services [19]. The path to universal health coverage and achieving the health-related Sustainable Development Goals (SDGs) will require countries around the world to train more frontline health workers to compensate for the attrition of existing healthcare workers through migration, retirement or death [7, 23]. For developing countries in particular, increasing the production of healthcare workers in disadvantaged areas/states will be required to address the current shortages, in addition to deploying strategies to address the maldistribution of health workers so as to facilitate a more equitable distribution of health workers [including key frontline health workers] to meet the health systems’ needs.

Data from this study broadly confirm the key steps for student enrolment into health training institutions to include – the sale of forms and the application process for prospective students, the selection of students, registration and then orientation/induction for newly admitted students. Stakeholders from health training institutions, Ministries of Health and other government agencies indicated that the actual student enrolment process can be complex and sometimes rather challenging to manage**.** It is however commendable that regulatory bodies hold regular discussions with health training institutions in Nigeria and provide the necessary guidance on the number of students who should be indexed each year based on criteria such as the accreditation status of heath training institutions, the available infrastructure and the number of tutors—this culture of constant engagement should be sustained.

Study participants’ descriptions of the application process were consistent—applicants purchase application forms, then complete and submit the application forms, before taking part in entrance examinations and oral interviews. Many stakeholders report that currently the content and delivery of entrance examinations for students into health training institutions are *fit-for-purpose*, but much more should be done for example, by the decentralization of entrance examination venues for applicants to reduce the stress felt by prospective students and their families. It is also noteworthy that the use of ‘weeding examinations’ is becoming standard practice (particularly in Bauchi state) to ensure that students of the right quality are indexed as trainee frontline health workers with the regulatory bodies. The use of ‘weeding examinations’ should be adopted as part of national guidelines for student enrolment into health training institutions across Nigeria [24]. Many study participants agree that the use of ‘weeding examinations’ serves as a valid quality assurance mechanism which helps to address challenges associated with ‘incompetent students’ as well as the negative influences of political interference and favouritism.

A major challenge identified, especially by students, was the high costs of tuition, registration fees and the cost of completing training programmes within health training institutions, which is compounded by the lack of financial support for students. The majority of stakeholders and students involved in this study emphasized the usefulness of scholarships for students but lamented that these were not available to students enrolled in Schools/Colleges of Nursing and Midwifery as well as Colleges of Health Technology, despite the availability of scholarships for other health professionals such as doctors. In more developed countries, there have been calls in the past for nurses to be supported with bursaries and possibly excluded from paying tuition fees during their training [25]. There have also been proposals for governments to subsidize nursing education especially in areas where nurses are underproduced in comparison to health system needs [7]. The Nigerian government should strongly consider providing bursaries to support students who enrol into health training institutions to become frontline health workers. Elected officials can also take up sponsoring student nurses, midwives and community health workers as part of their ‘constituency projects’ to promote human resource development for the health sector and increase the number of frontline healthcare workers [24]. In addition, all fees and any other charges should be transparently outlined in a prospectus and given to students as well as made available to the general public so that it is clear what the financial burden for students and their families will be as they plan to enroll into health training institutions [24].

Information-sharing to prospective students should be enhanced using as many communication channels as possible. Moreover, other channels for publicity such as using churches, mosques and community announcements maybe currently underutilized and should be better utilized in raising awareness about the student enrolment process. An increase in the use of social media (e.g. Facebook, Twitter and WhatsApp) for publicity which is increasingly being used for health promotion [26] and which most young applicants will likely engage with, could possibly also help increase information sharing and publicity for the student enrolment process. More emphasis should be placed on the appropriate use of new technologies as these are increasingly being used as part of the admission process in health training institutions around the world—this should be adopted and used in Nigerian health training institutions. The use of new technologies is part of recent innovations for the education of the health workforce [7].

There is need to strengthen the ‘bonding agreement process’ for students as well as ensure the prompt payment of the right amount of allowances to students by governments and other sponsoring bodies [24]. Going forward, it will be helpful to strengthen the trainee programme for student nurses and midwives in ways that guarantee the immediate employment of nurses and midwives into public service by the regulatory bodies working collaboratively with health training institutions and state governments. This approach of direct absorption of students (who have been indexed with the regulatory bodies) into public service should also be extended to community health worker programmes [24], with necessary adjustments for those desirous of working within the private health sector. In addition, much more advocacy should also be conducted to counter stereotypes which suggest that certain professions are exclusive for a particular gender [24].

To cope with the relatively high number of applicants competing for limited slots in health training institutions and to increase the number of frontline health workers produced within each state, state governments should consider establishing more Schools of Nursing and Midwifery and Colleges of Health Technology. State governments should possibly introduce and implement the collegiate system for health training institutions [27] just like Cross-River state is currently exploring. Amongst other benefits, the collegiate system will give rise to greater support for infrastructural development and maintenance from government agencies like the Tertiary Education Trust Fund (TETFUND)—as is applicable to other institutions of higher learning in Nigeria. This may be achieved by relevant officials within the Federal Ministries of Health and their counterparts in the Federal Ministry of Education working to integrate the infrastructural development and maintenance of health training institutions into the Tertiary Education Trust Fund using appropriate policy instruments and frameworks [24]. There should be concerted efforts to build, renovate and upgrade the infrastructure of health training institutions in Nigeria (including hostel accommodation for students, staff quarters for tutors, fencing of premises, etc.) An effective infrastructural development and maintenance strategy for health training institutions should also be developed with a feasible implementation framework.

The management of health training institutions should work with the Ministry of Health in each state to secure and sustain full accreditation from the relevant regulatory bodies, which will guarantee that the maximum number of students can be enrolled into these health training institutions. A national policy framework for the student enrolment of key cadres of frontline health workers should be developed to address Nigeria’s health workforce needs. This should be facilitated by collaboration between the National Primary Healthcare Development Agency (NPHCDA), Federal Ministry of Health and the relevant regulatory bodies. Different states in Nigeria should subsequently adapt the national policy framework for student enrolment and develop specific guidelines for student enrolments for their respective states, taking into consideration the specific context of each state [24].

## Conclusion

This study has provided detailed evidence about student enrolment processes, practices and challenges with respect to health training institutions in Bauchi and Cross-River States, which are largely reflective of the wider health system in Nigeria. The strategies and recommendations should be carefully considered and implemented by policy-makers in government, as well as different stakeholders across Ministries of Health, regulatory bodies and health training institutions. These strategies could have far-reaching implications for improving the quality and quantity of the frontline health workforce as well as enhance efforts at achieving universal health coverage.

## Data Availability

The datasets for this study are available from the corresponding author upon reasonable request.

## Abbreviations

CHEW: Community health extension worker
CHO: Community health officer
FLHW: Frontline health worker
FGD: Focus Group Discussion
GAC: Global Affairs Canada
HRH: Human resources for health
IRB: Institutional review board
JCHEW: Junior community health extension worker
LGA: Local government area
MMR: Maternal mortality rate
MNCH: Maternal newborn and child health
PHC: Primary health care
PHCUOR: Primary healthcare under one roof
WHO: World Health Organization

## References

[1] World Health Organization (2006). The World Health Report 2006: Working Together for Health.

[2] Appiagyei AA, Kiriinya RN, Gross JM (2014). Informing the scale-up of Kenya’s nursing workforce: a mixed methods study of factors affecting pre-service training capacity and production. Human Res Health.

[3] World Health Organization, GHWA: Report on the Prince Mahidol Award Conference 2011 and Second Global Forum on Human Resources for Health; 2011. http://www.who.int/workforcealliance/knowledge/resources/SecondHRHForum_report_en.pdf.. Accessed 24 June 2020.

[4] World Health Organization: Transforming and scaling up health professionals education and training: World Health Organization guidelines 2013; 2013. http://whoeducationguidelines.org/sites/default/files/uploads/WHO_EduGuidelines_20131202high_print.pdf.. Accessed 24 June 2020. 26042324

[5] Ezeonwu MC (2013). Nursing education and workforce development: Implications for maternal health in Anambra State Nigeria. Int J Nurs Midwifery.

[6] Nkwo PO, Lawani LO, Ubesie AC, Onodugo VA, Obu HA, Chinawa JM (2015). Poor availability of skilled birth attendants in Nigeria: a case study of Enugu state primary health care system. Ann Med Health Sci Res.

[7] World Health Organization. State of the world's nursing 2020: investing in education, jobs and leadership. Geneva: World Health Organization; 2020. https://www.who.int/publications/i/item/9789240003279 . Accessed 24 June 2020.

[8] National Primary Healthcare Development Agency (2012). National guidelines for development of primary healthcare system in Nigeria.

[9] Okereke E, Ishaku SM, Unumeri G, Mohammed B, Ahonsi B (2019). Reducing maternal and newborn mortality in Nigeria—a qualitative study of stakeholders’ perceptions about the performance of community health workers and the introduction of community midwifery at primary healthcare level. Hum Resour Health.

[10] Adegoke AA, Mani S, Abubakar A, van den Broek N (2013). Capacity building of skilled birth attendants: a review of pre-service education curricula. Midwifery.

[11] World Health Organization. Global Health Observatory data repository*.* World Health Organization. https://apps.who.int/gho/data/node.main.HWF. Accessed 5th Oct 2020.

[12] Adindu A, Asuquo A (2013). Training human resource for 21st century Nigerian health sector. Glob J Human Res Manag.

[13] Adeloye D, David RA, Olaogun AA, Auta A, Adesokan A, Gadanya M, Opele JK, Owagbemi O, Iseolorunkanmi A (2017). Health workforce and governance: the crisis in Nigeria. Human Res Health.

[14] Bangdiwala SI, Fonn S, Okoye O, Tollman S (2010). Workforce resources for health in developing countries. Public Health Rev.

[15] Gyuse AN, Ayuk AE, Okeke MC (2018). Facilitators and barriers to effective primary health care in Nigeria. Afr J Prim Health Care Fam Med.

[16] National Population Commission (NPC) [Nigeria] and ICF (2019). Nigeria demographic and health survey 2018.

[17] Eboreime EA, Abimbola S, Obi FA, Ebirim O, Olubajo O, Eyles J, Nxumalo NL, Mambulu FN (2017). Evaluating the sub-national fidelity of national initiatives in decentralized health systems: integrated primary healthcare governance in Nigeria. BMC Health Serv Res.

[18] Federal Government of Nigeria (2018). Nigeria’s national strategic health development plan (2018–2022).

[19] Sousa A, Scheffler RM, Nyoni J, Boerma T (2013). A comprehensive health labour market framework for universal health coverage. Bull World Health Organ.

[20] Ekechi O, Ibrahim S, Aisha J (2019). Strengthening Bauchi State College of Nursing and Midwifery by updating its training curricula, procedure manuals and student handbooks. HRH Project brief 2019.

[21] Ekechi O, Godwin U, Aisha J (2019). Strengthening Cross River state schools of nursing and midwifery by updating their training curricula, procedure manuals and student handbooks. HRH Project brief 2019.

[22] Abushaikha L (2016). Midwifery students’ enrolment reasons and evaluations of the first Bachelor of Midwifery programme in Jordan. Midwifery.

[23] Castro Lopes S, Guerra-Arias M, Buchan J, Pozo-Martin F, Nove A (2017). A rapid review of the rate of attrition from the health workforce. Hum Resour Health.

[24] Okereke Ekechi, Dirisu Osasuyi, Akinola Akinwumi, Unumeri Godwin, Suleiman Ibrahim, Jibril Aisha. An assessment of Student Enrolment and Tutor Recruitment Processes and Practices within health training institutions in Cross River and Bauchi States, Nigeria. Human Resources for Health (HRH) project 2019: Population Council. Unpublished Report.

[25] Nazarko L (2016). Scrapping bursaries: we must learn from the past. Br J Nurs.

[26] Neiger BL, Thackeray R, Van Wagenen SA, Hanson CL, West JH, Barnes MD, Fagen MC (2012). Use of social media in health promotion: purposes, key performance indicators, and evaluation metrics. Health Promot Pract.

[27] Hou J, Michaud C, Li Z, Dong Z, Sun B, Zhang J, Cao D, Wan X, Zeng C, Wei B, Tao L, Li X, Wang W, Lu Y, Xia X, Guo G, Zhang Z, Cao Y, Guan Y, Meng Q, Wang Q, Zhao Y, Liu H, Ke Y, Chen L (2014). Transformation of the education of health professionals in China: progress and challenges. Lancet.

